# Supplementary material for: Phenotypic and Molecular Characterization of Carbapenem-Heteroresistant Bacteroides fragilis Strains
Source: Antibiotics (Basel). 2022 Apr 27;11(5):590. doi: 10.3390/antibiotics11050590 (PMC9138018; doi:10.3390/antibiotics11050590)
Supplement: Supplementary file 1 [file antibiotics-11-00590-s001.zip › antibiotics-1684959-supplementary.pdf]

Supplementary Materials

Table S1. PCR experiment parameters

Primer	PCR type	Sequence 5’-3’	PCR cycling[a]	Ref.
cfiA1	conv.[b]	TCCATGCTTTTCCCTGTCGCAGTTAT	94 °C 30 s, 50 °C 1 min, 72 °C 1 min, 35x	
cfiA2		GGGCTATGGCTTTGAAGTGC		
Up2	conv.	TACGCTTTTCTGTGCCATAACTGC	94 °C 30 s, 52 °C 1 min, 72 °C 3 min,35x	
G		CGCCAAGCTTTGCCTGCCATTA		
gap-F	qRT-PCR	AGCCATTGTAGCAGCTTTTT		
gap-R		GAAGACGGGATGATGTTTTC		
cfiA-RT1	qRT-PCR	AATCGAAGGATGGGGTATGG		
cfiA-RT2		CGGTCAGTGAATCGGTGAAT	94 °C 15 s, 55 °C 30 s, 72°C 30s, 35x	This study
GNAT-F	qRT-PCR	ACAGAAATGGTGGAAGAAAT		
GNAT-R		GTTGACGGTAATCGTCTCTG		
XAT-F	qRT-PCR	CTGATAATCGGCAAGTTTTG		
XAT-R		CTTCGTAACCGATCCATACA		
Lrp-F	conv.	GAGGGGCTTGCGGCTGTG	94°C 30 s, 50°C 30 s, 72°C 30 s, 35x	This study
Lrp-R		ATCTTATGGTTGTTTTTCCG		

[a] For conventional PCR, we used starting denaturation and final elongation with the following parameters 94 °C 5 min and 72 °C 10 min, respectively. For qRT-PCR the values recommended by the supplier were used. [b] Conventional PCR.

Figure S1. Alignment of amino acid sequences of GNAT acetylating toxin homologs.

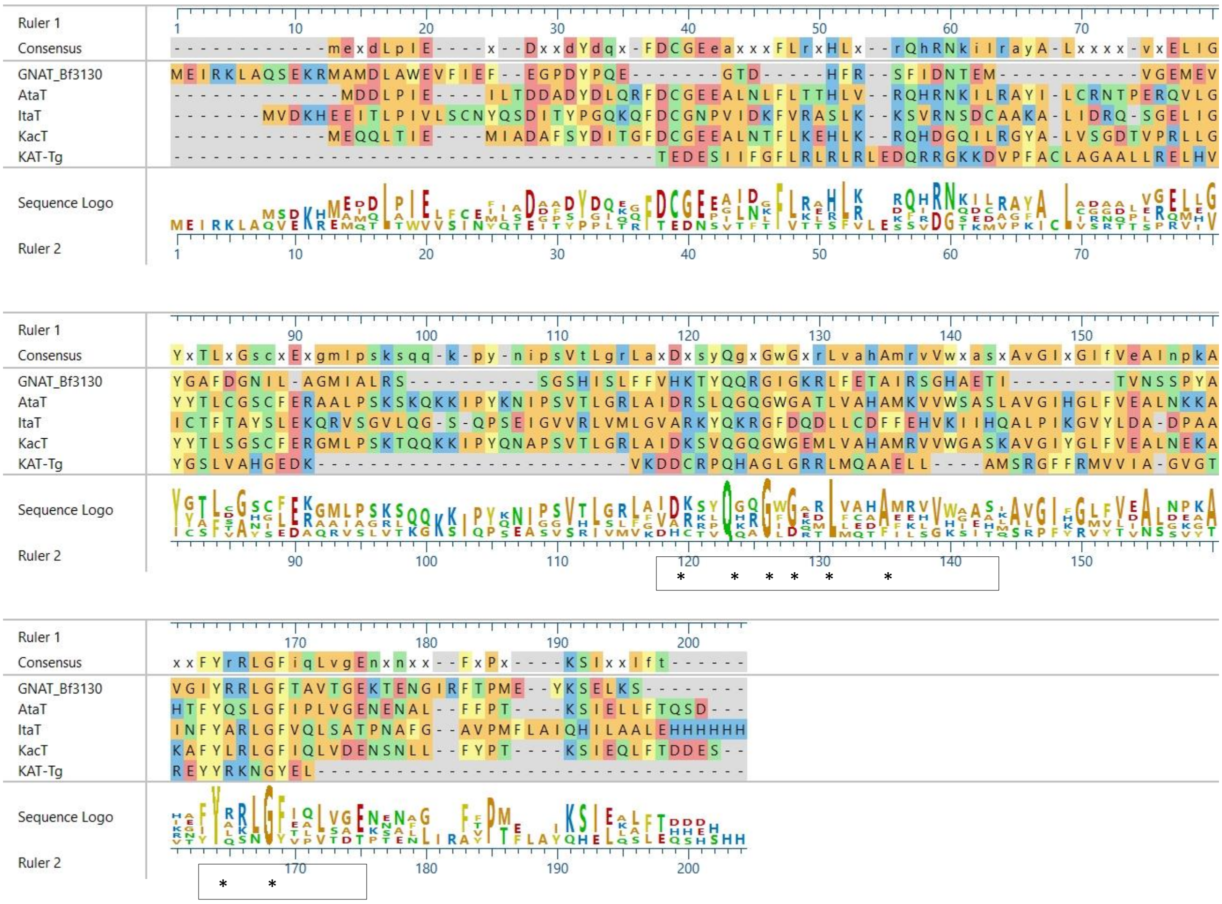

GNAT_Bf3130 – the ‘GNAT’ protein of the ‘*cfiA* element’ in *B. fragilis* 3130; AtaT – TA toxin of *Escherichia coli* (acc. no. WP_142447164); ItaT – TA toxin of *Escherichia coli* (acc. no. 7BYY_D); KacT – TA toxin of *Klebsiella pneumoniae* (acc. no QXW56992); KAT-Tg – the lysine acetylase domain in *Toxoplasma gondii* Elongation protein 3 (acc. no. PUA86011, residues 617-721)
Conserved active centre residues are marked with asterisks.

Table S2. Etest MIC values of the test strains

B. fragilis	Ref.	MICs[b]											
		AMP[c]	***AMC***	***FOX***	***IPM***	***MPM***	ERY	CLM	***MOX***	MTZ	TET	TIG	CHL
Susceptible controls													
NCTC 9343	-	16	0.25	4	0.032	0.064	8	1	0.125	0.125	0.125	0.125	4
638R	-	2	0.25	0.064	0.032	0.032	0.5	0.25	0.25	0.125	0.064	0.032	2
D39		**8**[d]	0.25	4	0.064	0.064	**16**	0.25	0.25	0.5	**16**	0.25	8
Silent/HR[a]													
7979	This study	**>256**	2	32	0.125	2	**16**	2	0.125	0.25	0.25	0.125	4
3130	This study	2	1	16	0.125	**8-(32)**	**8**	0.5	0.064	0.125	0.25	0.064	4
3035		1	1	8	0.125-(4)	2	**128**	2	0.125	0.25	8	0.25	4
SY69		1	1	16	0.25-(8)	**2-(32)**	**16**	2	2	0.125	8	0.125	4
CZE65		**32**		32	0.25-(1)	**2-(8)**	4	1	2	0.25	0.125	0.064	4
CZE60		**32-(128)**[e]	8	32	**1-(32)**	**>32**	**1-(8)**	1	**4-(16)**	0.25	0.125	0.064	**16**
SLO8		**>256**	8	**128**	**4-(>32)**	**>32**	2	1	0.125	0.25	0.125	0.25	4
HR-ind[a]													
3130i5	This study	**8**	2	16	**0.5-(16)**	**>32**	**16**	1	0.064	0.032	0.125	0.064	4
CZE60i	This study	**>256**	8	**64-(256)**	**>32 (4-(256)[f])**	**>32**	1	1	2	0.25	0.125	0.064	**16**
With IS[a]													
De248514/19	This study	**32**	4	32	**1-(8)**	**>32**	**8-(256)**	2	0.125	0.125	**16**	0.5	4
1672		**>256**	8	**128**	**>32 (8-(128)[f])**	**>32**	**8-(32)**	2	0.25	0.5-(2)	**16**	1	8
TAL3636		**>256**	**4-(16)**	**128**	**>32 (256[f])**	**>32**	4	2	0.064	0.125	4	0.125	4

[a] Silent/HR – silently or heterogeneously resistant, HR-ind – induced heteroresistant, with IS – IS element in the upstream region of the *cfiA* gene. [b] µg/ml. [c] AMP – ampicillin, AMC – amoxicillin/clavulanic acid, FOX – cefoxitin, IPM – imipenem, MPM – meropenem, ERY – erythromycin, CLM – clindamycin, MOX – moxifloxacin, MTZ – metronidazole, TET – tetracycline, TIG – tigecycline, CHL – chloramphenicol (bactericidal types are bold italic). [d] Resistant values are shown in bold. [e] Heteroresistance is shown by rust colour. [f] The IPM MICs were from MBL Etests.

Table S3. Lysine-rich peptide sequences of the *cfiA*-positive *B. fragilis* strains

Strain	Amino acid and nucleotide sequence[a]	Number of lysines and (ratio)
B. fragilis		
7979	M L R D H **K** R **K** **K** N **K** Q D I D G T R T Y **K** **K** **K** Y H Y<br>atgcttagagatcataaaagaaagaaaaacaaacaggatatagacggcacacgaacttataaaaagaagtatcattactaa	7 (26.9 %)
3130	M L G N H **K** R E A N S I Q T A H E L I **K** R S I I T **K** L S Q H R **K** N N H **K** I<br>atgcttgggaatcataaaagagaggcaaacagtatccagacggcacacgaacttataaaaagaagtatcattactaaactttcccaacatcggaaaaacaaccataagatataa	5 (13.5 %)
3035	M L R D H **K** R **K** **K** **K** **K** Q Y P D G T R T Y **K** **K** **K** Y H Y Q T F P P S E **K** Q P<br>atgcttagagatcataaaagaaagaaaaagaaacagtatccagacggcacacgaacttataaaaagaagtatcattaccaaactttcccaccatcggaaaaacaaccataa	9 (25.0%)
SY69	M L G N H **K** R E A N S I Q T A H E L I **K** R S I I T **K** L S Q H R **K** N N H **K** I<br>atgcttgggaatcataaaagagaggcaaacagtatccagacggcacacgaacttataaaaagaagtatcattactaaactttcccaacatcggaaaaacaaccataagatataa	5 (13.5 %)
CZE65	M L R D H **K** R **K** **K** N **K** Q D I D G T R T Y **K** **K** **K** Y H Y<br>atgcttagagatcataaaagaaagaaaaacaaacaggatatagacggcacacgaacttataaaaagaagtatcattactaa	7 (26.9 %)
CZE60	M L R D H **K** R **K** **K** N **K** Q D I D G T R T Y **K** **K** **K** Y H Y<br>atgcttagagatcataaaagaaagaaaaacaaacaggatatagacggcacacgaacttataaaaagaagtatcattactaa	7 (26.9 %)
SLO8	M L R D H **K** R **K** **K** **K** **K** Q Y P D G T R T Y **K** **K** **K** Y H Y Q T F P P S E **K** Q P<br>atgcttagagatcataaaagaaagaaaaagaaacagtatccagacggcacacgaacttataaaaagaagtatcattaccaaactttcccaccatcggaaaaacaaccataa	9 (25.0 %)
3130i5	M L G N H **K** R E A N S I Q T A H E L I **K** R S I I T **K** L S Q H R **K** N N H **K** I<br>atgcttgggaatcataaaagagaggcaaacagtatccagacggcacacgaacttataaaaagaagtatcattactaaactttcccaacatcggaaaaacaaccataagatataa	5 (13.5 %)
CZE60i	M L R D H **K** R **K** **K** N **K** Q D I D G T R T Y **K** **K** **K** Y H Y<br>atgcttagagatcataaaagaaagaaaaacaaacaggatatagacggcacacgaacttataaaaagaagtatcattactaa	7 (26.9 %)
De248514	M L R D H **K** R **K** **K** **K** **K** Q Y P D G T R T Y **K** **K** **K** Y H Y Q T F P P S E **K** Q P<br>atgcttagagatcataaaagaaagaaaaagaaacagtatccagacggcacacgaacttataaaaagaagtatcattaccaaactttcccaccatcggaaaaacaaccataa	9 (25.0 %)
1672	M L R D H **K** R **K** **K** N **K** Q D I D G T R T Y **K** **K** **K** Y H Y<br>atgcttagagatcataaaagaaagaaaaacaaacaggatatagacggcacacgaacttataaaaagaagtatcattactaa	7 (26.9 %)
TAL3636	M L R D H **K** R **K** **K** **K** **K** Q Y P D G T R T Y **K** **K** **K** Y H Y Q T F P P S E **K** Q P<br>atgcttagagatcataaaagaaagaaaaagaaacagtatccagacggcacacgaacttataaaaagaagtatcattaccaaactttcccaccatcggaaaaacaaccataa	9 (25.0 %)

[a] Lysines (K) are shown in bold.

Figure S2. Showing the variations in the alignment of lysine-rich peptide nucleotide sequences in case of each sequence types

```
Lrp_3130      atgcttggg----aatcataaaagagaggcaaacagtatccagacggcacacgaacttat  56
Lrp_CZE60     atgcttagagatcataaaagaaagaaaaacaaacaggatatagacggcacacgaacttat  60
Lrp_De248514  atgcttagagatcataaaagaaagaaaaagaaacagtatccagacggcacacgaacttat  60
              ****** *     *   *  ***** *   ****** **  *******************

Lrp_3130      aaaaagaagtatcattactaaactttcccaacatcggaaaaacaaccataagat-ataa   114
Lrp_CZE60     aaaaagaagtatcattactaaactttcccaacatcggaaaaacaaccataagat-atag   118
Lrp_De248514  aaaaagaagtatcattaccaaactttcccaccatcggaaaaacaaccataagatcctaa   119
              ****************** *********** ***********************  **
```

Start codons are underlined, stop codons are bold underlined. Aligning nucleotides are marked with asterics
